# Local T-Cell Dysregulation and Immune Checkpoint Expression in Human Papillomavirus-Mediated Recurrent Respiratory Papillomatosis

**DOI:** 10.3390/cells14130985

**Published:** 2025-06-27

**Authors:** Hans N. C. Eckel, Su Ir Lyu, Frederik Faste, Shachi J. Sharma, Anne Nobis, Nora Wuerdemann, Maria Ziogas, Marcel Mayer, Malte C. Suchan, Kerstin Wennhold, Maria A. Garcia-Marquez, Martin Thelen, Elena Hagen, Julia Eßer, Charlotte Klasen, Oliver Siefer, Martin Otte, Hans A. Schloesser, Jens P. Klussmann, Alexander Quaas, Kevin K. Hansen

**Affiliations:** 1 Faculty of Medicine and University Hospital of Cologne, Department of Otorhinolaryngology, Head and Neck Surgery, University of Cologne, 50931 Cologne, Germanykevin.hansen@uk-koeln.de (K.K.H.); 2 Center for Molecular Medicine Cologne, Faculty of Medicine and University Hospital of Cologne, University of Cologne, 50931 Cologne, Germany; nora.wuerdemann@uk-koeln.de (N.W.); martin.thelen@uk-koeln.de (M.T.);; 3 Faculty of Medicine and University Hospital of Cologne, Institute of Pathology, University of Cologne, 50937 Cologne, Germany; 4 Faculty of Medicine and University Hospital Cologne, Department I of Internal Medicine, Center for Integrated Oncology Aachen Bonn Cologne Duesseldorf, University of Cologne, 50937 Cologne, Germany; 5 Faculty of Medicine and University Hospital of Cologne, Department of General, Visceral, Cancer and Transplantation Surgery, University of Cologne, 50937 Cologne, Germany

**Keywords:** human papillomavirus, recurrent respiratory papillomatosis, lymphocytes, CTLA4

## Abstract

Human papillomavirus-mediated recurrent respiratory papillomatosis (RRP) is a premalignant neoplasia of the upper airway characterized by significant dysphonia and respiratory obstruction. Immune checkpoint blockade has emerged as a potential alternative to repeated surgical interventions in RRP. Here, we investigated the intralesional T-cell composition and expression of the immune checkpoints programmed death-ligand 1 (PD-L1) and cytotoxic T-lymphocyte antigen 4 (CTLA-4) in RRP. We analyzed tissue samples from 30 patients treated at a tertiary care center between 2009 and 2021, including paired samples from individual patients collected at different time points. Immunohistochemical staining was performed for CD4, CD8, CTLA-4, FoxP3, and PD-L1 and correlated with disease severity and previous adjuvant therapies. Overall disease burden and intervention-free survival were not associated with the abundance of CD4^+^, CD8^+^, or FoxP3^+^ T cells, nor with immune checkpoint expression. However, patients with aggressive disease exhibited a higher intralesional FoxP3/CD4 T-cell ratio. Prior intralesional cidofovir treatment was associated with reduced CD4^+^ T-cell infiltration. These findings suggest that a locally immunosuppressive microenvironment, reflected by an elevated FoxP3/CD4 ratio, contributes to disease severity in RRP. Consistent CTLA-4 expression across all evaluated samples supports further investigation of anti-CTLA-4 therapy, either alone or in combination with other checkpoint inhibitors.

## 1. Introduction

Recurrent respiratory papillomatosis (RRP) is characterized by the frequent recurrence of benign neoplasms within the respiratory tract caused by mucosal human papillomavirus (HPV) infection. HPVs, double-stranded DNA viruses, establish chronic mucosal infections but rarely cause systemic spread [1]. RRP is most commonly associated with low-risk HPV types 6 and 11, although high-risk HPV 16, 18, 31 and 33 may also be detected in papilloma tissue, albeit less frequently [2]. Canonically, the onset of RRP is bimodal, with two distinct incidence peaks: the first peak in children, typically around age 5, and the second peak in adults, around age 30. Recent data suggest a third peak later in life, at age 64 [3,4,5]. While RRP is generally considered benign, Freeman et al. reported malignant degeneration in 16.3% of smokers and 3.6% of nonsmokers, with other reports estimating the occurrence of malignant transformation in 3 to 7% of RRP cases, classifying RRP as a premalignant condition [6,7,8]. Although RRP primarily affects the upper airways, particularly the membranous vocal folds and false vocal cords, it can spread throughout the respiratory tract, with rare involvement of the trachea, nasopharynx, and lung [9,10,11,12].

Patients with RRP exhibit impaired immune responses to HPV-6 and -11, including preferential differentiation of CD4^+^ T cells towards T helper 2 lineage and dysfunction in natural killer cells [13]. This may be partly driven by HPV-mediated inhibition of Langerhans cell activation and maturation, resulting in an impaired HPV-specific Th1 response [14]. Furthermore, increased levels of immune checkpoint molecules such as programmed-death ligand 1 and 2 (PD-L1 and PD-L2)—as well as the presence of T-cells expressing programmed cell death protein 1 (PD-1), T-cell immunoreceptor with Ig and ITIM domains (TIGIT), lymphocyte-activation gene 3 (LAG3), and T-cell immunoglobulin and mucin-domain containing-3 (TIM3)—have been described within the RRP lesions [15,16,17]. In summary, RRP is characterized by an immunosuppressive microenvironment with increased expression of immune checkpoint molecules and immunosuppressive cytokines, as well as a marked absence of interferon-gamma (IFN-γ) expression [18,19].

Careful surgical debulking remains the primary treatment for RRP, with options such as CO_2_-laser surgery and microdebrider-assisted surgical excision commonly employed to maintain airway patency and minimize mucosal damage [20,21,22]. However, due to frequent recurrence, many patients require multiple surgeries to maintain glottic function. Therefore, adjuvant therapies aiming to increase the intervals between surgeries have been tested [23,24,25]. Immune checkpoint blockade (ICB) has emerged as a systemic therapy for hematologic and solid malignancies and provides substantial benefits for patients [26,27]. A phase II study of the PD-L1 inhibitor avelumab in patients with aggressive RRP demonstrated safety and clinical activity, with a significant number of patients showing improvement in disease burden and a reduction in the frequency of surgical interventions, with similar results reported for nivolumab, a PD-1 inhibitor [28,29].

As RRP is a rare but devastating disease, with some patients requiring over 100 procedures in extreme cases, further investigation into novel treatment modalities, including ICB, is warranted to improve patient outcomes and reduce healthcare costs. In this study, we analyzed the T-cell composition and expression of immune checkpoints PD-L1 and cytotoxic lymphocyte antigen 4 (CTLA4), a co-inhibitory molecule not previously investigated in RRP, in a representative cohort of RRP patients in a German tertiary care center.

## 2. Materials and Methods

### 2.1. Patient Population and Data Collection

We conducted a retrospective study on a cohort of 30 RRP patients treated and followed up at the University of Cologne. The inclusion criteria for this study were the availability of RRP tissue specimens and available clinical data in patient records. Patients with a histological diagnosis of papilloma and positive HPV 6 or 11 DNA status were included. After reviewing the medical records and availability of RRP tissue specimens of patients with histologically confirmed laryngeal papillomatosis treated between 2009 and 2021, 38 patients were eligible. Of these patients, 30 provided written informed consent and were included in this study. The institutional review board of the University of Cologne approved this study (Review No. 21-1256, dated 17 August 2021). Clinical data collected from medical records included sex, age at initial diagnosis of RRP, lifetime number of needed surgical interventions, tracheal involvement, use of intralesional adjuvant treatment, and HPV vaccination status.

### 2.2. HPV-DNA Genotyping

HPV-DNA genotyping was performed for all patients using the HPV 3.5 LCD-Array (Chipron GmbH, Berlin, Germany). Extracted DNA was analyzed for the presence of HPV genotypes (6, 11, 16, 18, 31, 33, 35, 39, 42, 44, 45, 51, 52, 53, 54, 56, 58, 59, 61, 62, 66, 67, 68, 70, 72, 73, 81, 82, 83, 84, 90, 91) using a PCR-based approach, targeting conserved regions of the viral genome. The resulting PCR products were then subjected to hybridization on the HPV 3.5 LCD-Array, followed by a secondary labeling step and subsequent signal detection according to the manufacturer’s instructions. After washing and drying, the arrays were scanned and analyzed using a dedicated imaging system for pattern recognition and genotype identification.

### 2.3. Immunohistochemistry and Digital Evaluation

Immunohistochemical stainings were carried out in alignment with the manufacturers’ guidelines. The staining panel included CD4, CD8, CTLA4, FoxP3, and PDL1. Additional information about the antibodies is available in Supplementary Table S1. All stainings were performed using the automated Leica BOND-MAX system (Leica Biosystems, Wetzlar, Germany) following the recommended protocols. The resulting slides were digitized with the Aperio GT 450 DX scanner (Leica Biosystems, Wetzlar, Germany). Digital analysis of the stainings was conducted using QuPath v0.3.2, with a customized script adapted from Bankhead et al. [30]. For each slide, ten randomized 250 × 250 µm fields within the papilloma region were selected. Using a trained pixel classifier, the analyzed regions were segmented into epithelium, stroma, and background. Within these classified areas, cells were detected and classified within these compartments to enable independent quantification of positive and negative cells.

### 2.4. Statistical Analysis

Data processing, statistical analysis, and visualization were conducted using GraphPad Prism version 10.4.0. The differences between individual groups were assessed for significance using the nonparametric Mann–Whitney test. For the RRP cohort, intervention-free survival was defined as time between two surgical interventions. Kaplan–Meier curves were generated, with significance determined by the log-rank test. Patients without events or lost to follow-up were censored at the last known date. *p* values < 0.05 were considered significant.

## 3. Results

The study population included a cohort of 30 patients with RRP; 17 (57%) patients were male, while 13 (43%) were female. The median age of the patients at disease onset was 32 years (range 1–61); considering patients below the age of 12 years (juvenile-onset), 4 patients were classified as juvenile-onset, while 26 patients had adult-onset RRP. Fifteen (50%) patients had received intralesional injection of either bevacizumab or cidofovir, and six (20%) had received HPV vaccination (either Gardasil^®^ or Gardasil 9^®^). Patient characteristics are displayed in Table 1.

### 3.1. FOXP3^+^/CD4^+^ T-Cell Ratio (But Not Total T-Cell Infiltration or Immune Checkpoint Expression) Is Related to Clinical Disease Severity

**Figure 1:**
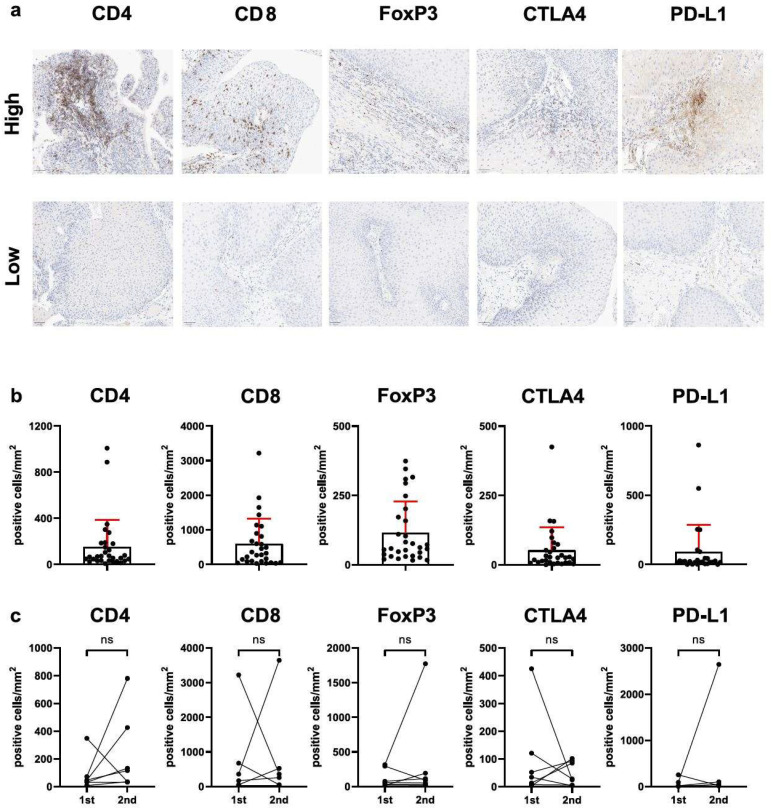
CD4-, CD8-, FoxP3-, CTLA4-, and PD-L1-positive immune cells infiltrate recurrent respiratory papillomatosis (RRP) tissue. Tissue samples of patients with RRP were stained for the indicated markers by immunohistochemistry. (**a**) Representative images of tissue samples with high and low expression of the indicated molecules. (**b**) Number of positively stained cells/mm^2^ displayed as individual values, mean, and standard deviation (*n* = 30). (**c**) CD4-, CD8-, FoxP3-, CTLA-4- and PD-L1-positive cells/mm^2^ in consecutive surgical specimens (*n* = 7); ns, not significant; mm, millimeter. Significant differences were calculated by Mann–Whitney test.

T-cells are essential for both the immediate and long-term immune defense against HPV, functioning through direct cytotoxicity, helper activities, and the establishment of immunological memory. To elucidate the intralesional abundance of CD8^+^, CD4^+^, and FoxP3^+^ T-cells, we performed immunohistochemical staining of whole-slide RRP tissue specimens (Figure 1a,b). Furthermore, intralesional expression of inhibitory immune checkpoint molecules PD-L1 and CTLA4 was measured (Figure 1a,b). The mean infiltration of CD4^+^ T-cells was 153 ± 234 cells/mm^2^, while the mean infiltration of CD8^+^ T-cells was 596 ± 724 cells/mm^2^, and that for FoxP3^+^ cells was 116 ± 112 FoxP3^+^ cells/mm^2^ (Figure 1b). The mean number of cells with expression of CTLA4 was 52 ± 83 cells/mm^2^, and that for PD-L1 was 92 ± 194 cells/mm^2^. Notably, expression of CTLA4 and PD-L1 was detected in all tissue specimens. As tissue from multiple consecutive surgeries was available from seven patients, we compared T-cell abundance and immune checkpoint expression in consecutive samples (Figure 1c), but we found no significant differences.

**Figure 2:**
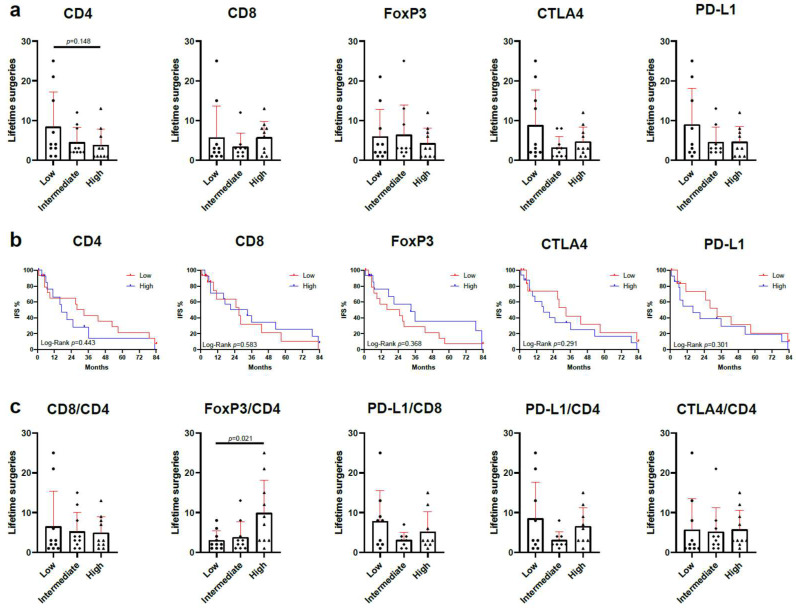
Correlation of T-cell abundance and immune checkpoint expression with clinical severity reveals more previous surgeries in patients with a high ratio of FoxP3^+^/CD4^+^ cells. Patients are divided into three groups—low, intermediate, and high—according to expression of the respective target molecules (*n* = 10 per group). (**a**) Number of previous lifetime surgeries of patients with respect to immune cell infiltration and immune checkpoint expression. (**b**) Intervention-free survival in relation to the expression of CD4, CD8, FoxP3, CTLA4, and PD-L1. (**c**) Lifetime surgeries in relation to the intralesional ratio of CD8^+^/CD4^+^ cells, FoxP3^+^/CD4^+^ cells, PD-L1^+^/CD8^+^ cells, PD-L1^+^/CD4^+^ cells, and CTLA4^+^/CD4^+^ cells. IFS, intervention-free survival. Significant differences were calculated by Mann–Whitney test (comparing low vs. high groups) and log-rank test.

Next, we evaluated the relation of T-cell infiltration and immune checkpoint expression to clinical disease severity. Abundance of CD4^+^, CD8^+^, and FoxP3^+^ T-cells and expression of immune checkpoints CTLA4 and PD-L1 were not associated with the number of surgeries patients had undergone previously (Figure 2a). We analyzed whether immune cell abundance or immune checkpoint expression would influence the intervention-free survival (IFS), defined as the time until the next surgical intervention for RRP is required. Intralesional abundance of CD4-, CD8-, FoxP3-, CTLA4-, or PD-L1-positive cells did not lead to a significantly improved IFS (Figure 2b). Furthermore, we evaluated the ratio of Foxp3^+^ cells to CD4^+^ cells. Patients with a low FoxP3/CD4 ratio had fewer previous surgeries compared to those with a high ratio (*p* = 0.021) (Figure 2c). Determining the ratios of CD8/CD4-, PD-L1/CD8-, PD-L1/CD4-, and CTLA4/CD4-positive cells did not provide additional stratification of patient risk based on T-cell infiltration or immune checkpoint expression.

### 3.2. Analysis of Stroma and Epithelium Reveals Special Distribution of Intralesional T-Cells

**Figure 3:**
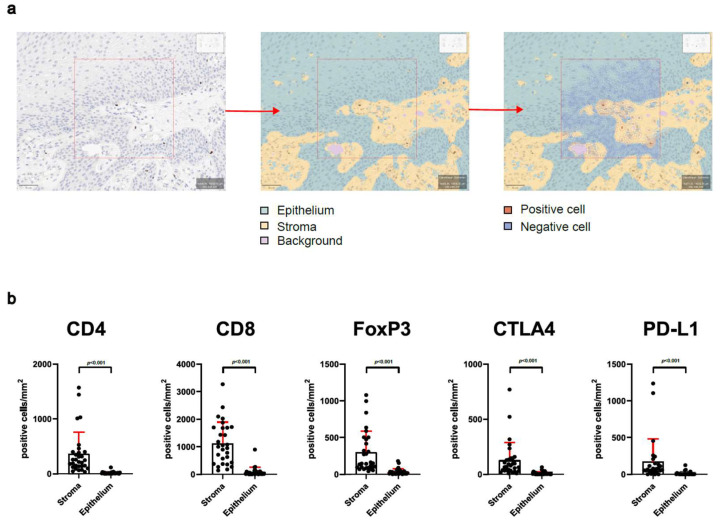
Immune cell infiltration is more abundant in stromal tissue than in epithelial tissue. (**a**) Workflow for the classification of recurrent respiratory papillomatosis tissue stroma and epithelium, as well as positive cell detection. (**b**) Investigation of the distribution of CD4-, CD8-, FoxP3-, CTLA4-, and PD-L1-positive cells within the stromal and epithelial compartments of RRP tissue; mm, millimeter. Statistical differences were calculated by Mann–Whitney test.

Furthermore, we analyzed whether the spatial distribution of infiltrating lymphocytes would influence the clinical course of RRP. Tissue sections were categorized as either stroma or epithelium, and infiltrating cells within these compartments were analyzed individually (Figure 3a). T-cell abundance and immune checkpoint expression were significantly higher in the stromal compartment compared to epithelium (*p* < 0.001) (Figure 3b). Immune cell abundance and immune checkpoint expression in RRP epithelium did not correlate with previous surgeries or IFS (Supplementary Figure S1a,b). In the stromal compartment, CD4^+^, CD8^+^, and FoxP3^+^ cells and PD-L1 and CTLA4 expression were not related to clinical disease severity (Supplementary Figure S1c,d).

### 3.3. Adjuvant Therapy with Cidofovir Is Related to Reduced CD4^+^ T-Cell Infiltration

**Figure 4:**
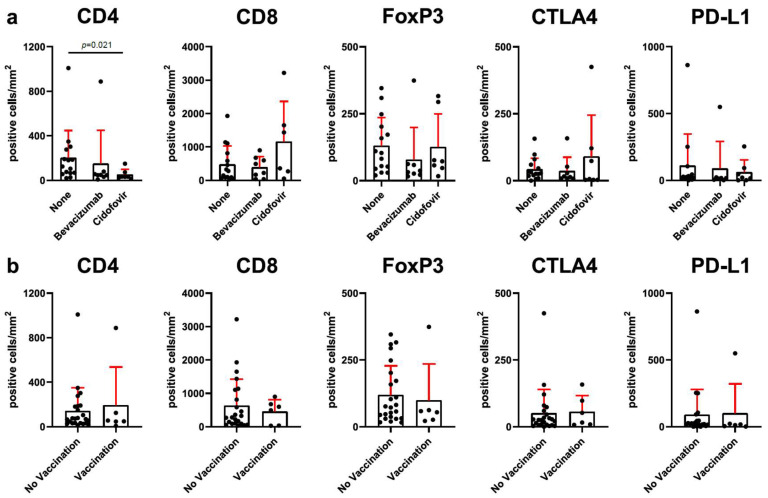
Cidofovir treatment influences CD4^+^ T-cell infiltration in RRP. Tissue samples of RRP patients were analyzed by immunohistochemistry for the expression of the indicated markers. (**a**) Samples without previous adjuvant therapy (*n* = 15) were compared to samples with either previous bevacizumab (*n* = 8) or cidofovir (*n* = 7); statistical differences were calculated by Mann–Whitney test (none vs. bevacizumab or cidofovir). (**b**) Prophylactic HPV vaccination (Gardasil^®^ or Gardasil 9 ^®^) (*n* = 6) compared to unvaccinated (*n* = 24) patients; significant differences were calculated by Mann–Whitney test.

In an effort to reduce the number of surgeries needed, many adjuvant therapies have been tested to treat RRP [31]. In our department, intralesional bevacizumab is routinely recommended for patients with high-risk disease, whereas cidofovir is no longer utilized due to its associated adverse effect profile. To investigate whether the efficacy of previous cidofovir or bevacizumab injections are related to a change in intralesional immune cell abundance, we compared patients who did not receive previous local adjuvant therapies with those who were injected with either bevacizumab or cidofovir (Figure 4a). Patients who previously received at least one cidofovir injection had a reduced CD4^+^ cell infiltration (51 ± 47 vs. 202 ± 245, *p* = 0.021), but the number of FoxP3^+^ and CD8^+^ cells did not differ. The expression of immune checkpoints CTLA4 and PD-L1 was not significantly different between the three groups. In some institutions, prophylactic HPV vaccines Gardasil^®^ or Gardasil9^®^ are recommended as adjuvant therapy for RRP patients. In our cohort, six patients (20%) were vaccinated prior to the surgical intervention. The comparison of CD4^+^, CD8^+^, and FoxP3^+^ cells and immune checkpoints CTLA4 and PD-L1 did not reveal a difference in relation to vaccination status (Figure 4b).

## 4. Discussion

With the clinical availability of immune checkpoint inhibitors, an interest in the expression and role of immune checkpoints in RRP has developed. Notably, while expression of PD-1 and PD-L1, as well as other immune checkpoints in RRP, has been reported [16,17], to the best of our knowledge, this is the first report that describes the expression of intralesional CTLA4 in RRP.

High counts of FoxP3^+^ regulatory T-cells (Tregs) are observed in other chronic viral infections of mucosal tissues, including human immunodeficiency viruses (HIV), indicative of a local immunosuppressive microenvironment [32]. Hatam et al. found that functional Tregs (CD4^+^FoxP3^+^) are markedly enriched in RRP lesions, with no significant association with clinical disease severity [18]. The presence of Tregs and Treg-promoting cytokines such as interleukin-4 (IL-4), IL-10, and transforming growth factor-β (TGF-β) was significantly higher in aggressive cases of juvenile-onset RRP. We report that patients with a low ratio of FoxP3/CD4-positive cells had a lower number of previous surgeries compared to patients with a high FoxP3/CD4 ratio. This discrepancy may be explained by the small sample sized included in both studies, the application of different detection methods (flow cytometry or immunohistochemistry), or regional differences (North America or Europe). In our cohort, the expression of PD-L1 and the ratio of PD-L1^+^/CD8^+^ and PD-L1^+^/CD4^+^ cells were not correlated to disease severity or intervention-free survival, consistent with previous reports [15,17]. Furthermore, our results concur with previous studies observing no association of individual intralesional infiltration of CD4^+^ or FoxP3^+^ T-cells with disease severity in RRP [17]. CD8^+^ T-cells play a crucial role in the ability of the immune system to fight virus-infected cells and cancer by releasing cytotoxic molecules such as perforin and granzymes, as well as cytokines like IFN-γ, upon the recognition of MHC I-bound peptide fragments. Notably, in contrast to a previous publication by Ahn et al., CD8^+^ TILs were not associated with disease severity in our cohort [17]. Possible explanations for this disparity include the limited sample size in both studies, heterogeneous patient populations and regional differences in prevalent HPV types between Europe and the USA [33]. Furthermore, a limitation of the presented data is the unavailability of co-staining of analyzed T-cell markers and immune checkpoint molecules on individual cells, which would have allowed for a more precise characterization of cell populations. Notably, while CD8 and CD4 are established markers of the respective T-cell populations, co-staining with CD3 is commonly employed in immunobiology to verify that positive cells are in fact T-cells, as CD8^+^/CD3^−^ and CD4^+^/CD3^−^ non-T-cell populations have been described even though there is no direct evidence of the presence of these cell populations in RRP [34,35].

Separate analyses of T-cell infiltration and immune checkpoint expression in the stromal and epithelial compartment of RRP revealed that the stroma hosts the vast majority of T-cell and immune checkpoint expression. Analyses of the two distinct compartments in RRP are sparsely available [36], but they did not reveal further correlation of T-cell infiltration or immune checkpoint expression with clinical disease severity.

Intralesional application of adjuvant therapies with cidofovir or bevacizumab has shown some efficacy in reducing the frequency of surgical interventions and improving disease control without significantly increasing the risk of dysplasia or malignant transformation in RRP [37,38]. Complementing a recent study published by Solarz et al. [36], we found a significantly lower infiltration of CD4^+^ T-cells in patients who previously received intralesional cidofovir injections compared to patients with no previous adjuvant therapies. No influence of HPV vaccination with Gardasil^®^ or Gardasil9^®^ on immune cell infiltration was detected. This may be due to the preventive nature of these vaccines as they target the L1 protein, but another factor is the low number of HPV-vaccinated patients present in our cohort. The factors contributing to the low vaccination rate in Germany are likely related to national vaccination policies, the relatively late inclusion of boys in vaccination recommendations, and a high level of vaccine skepticism in the general population.

Recent advances in pharmacologic therapies, including systemic bevacizumab, have complemented the standard treatment of RRP, which primarily relies on repeated surgical debulking of papillomatous lesions [39,40]. While systemic bevacizumab is an efficacious therapy for RRP, life-long treatment is needed. Immune checkpoint inhibitors have shown promising results in the treatment of recurrent respiratory papillomatosis. A phase II study demonstrated that avelumab, a PD-L1 inhibitor, was safe and showed clinical activity in patients with laryngeal RRP, with a significant reduction in the need for surgical interventions [28]. Notably, while anti-PD-L1 therapy has demonstrated clinical activity in patients with laryngeal RRP, as reported by C. Allen et al., all four patients presenting with pulmonary lesions included in this trial did not have a response of pulmonary RRP. One patient with both laryngeal and pulmonary disease demonstrated a response to avelumab therapy in the larynx but no response in the lung. This discordance may suggest a distinct biology of pulmonary manifestations of RRP mediating primary resistance to immune checkpoint therapy. Similar results have been achieved with the PD-1 inhibitors pembrolizumab and nivolumab, highlighting the therapeutic potential of immune checkpoint inhibition in RRP [29,41].

In cancer therapy, the combination of anti-CTLA4 and anti-PD-1 antibodies has demonstrated enhanced anti-tumor efficacy compared to monotherapy in various cancers, including melanoma and non-small-cell lung cancer [42,43]. Mechanistically, the combination therapy induces unique cellular responses that are not observed with monotherapies, marked by an increase in the frequency of activated, terminally differentiated effector CD8^+^ T-cells and T helper type 1 (Th1)-like CD4^+^ effector T-cells, which are crucial for effective anti-tumor responses [43]. In RRP, to date, neither anti-CTLA4 therapy alone or in combination with anti-PD-1/PD-L1 antibodies has been evaluated. This may be the case because anti-CTLA4 therapies, such as those involving ipilimumab, are associated with a higher risk of immune-related adverse events (irAEs) [44]. When anti-CTLA4 antibodies are combined with anti-PD-1 antibodies, the risk of severe irAEs is further increased [45]. However, multiple new CTLA4 antibodies aiming to reduce irAEs compared to existing anti-CTLA4 antibodies are in clinical development [46,47]. Regulatory approval of these new drugs may enable the application of anti-CTLA4 antibodies alone or in combination with anti-PD-1 antibodies for the treatment of RRP.

## 5. Conclusions

We observed that RRP patients with aggressive clinical disease had a higher intralesional ratio of FoxP3/CD4 T-cells. In addition, previous intralesional cidofovir application was related to reduced CD4 T-cell infiltration. Expression of CTLA4 in RRP tissue was detectable in all evaluated cases, providing evidence for the possible study of anti-CTLA4 checkpoint inhibition alone or in combination with other checkpoint inhibitors in RRP.

## Figures and Tables

**Table 1:** Patient characteristics and clinical demographics of the recurrent respiratory papillomatosis cohort. All data were collected retrospectively from patients with available tissue specimens; *n*, number; NA, not applicable.

Patient Characteristics		RRP Cohort *n* = 30 (100%)
Sex	Male	17 (57%)
	Female	13 (43%)
Age	Median (range)	32 (1–61)
Onset	Juvenile	4 (13%)
	Adult	26 (87%)
Surgical interventions	Median (range)	3 (1–21)
Previous adjuvant treatment	Yes	15 (50%)
	No	15 (50%)
HPV vaccination	Yes	6 (20%)
	No	24 (80%)
Involvement of trachea	Yes	2 (7%)
	No	28 (93%)

## Data Availability

Data are available from the corresponding author upon reasonable request.

## Supplementary Materials

The following supporting information can be downloaded at: https://www.mdpi.com/article/10.3390/cells14130985/s1, Figure S1: Individual analysis of epithelial and stromal compartments. Patients are divided into three groups—low, intermediate, and high—according to expression of the respective target molecules (*n* = 10 per group); (a) workflow for the classification of recurrent respiratory papillomatosis tissue and positive cell detection; investigation of the stromal compartment for the relation of CD4-, CD8-, FoxP3-, CTLA4-, and PD-L1-positive cells with (b) previous surgeries and (c) intervention-free survival; stromal infiltration in relation to lifetime surgeries (d) and intervention-free survival (e); IFS, intervention-free survival; *p* values calculated by Mann–Whitney test (low vs. high) or log-rank test.; Table S1: Information of antibodies applied for histological staining: A = adenoid; T = tonsil.

## Abbreviations

The following abbreviations are used in this manuscript:

RRP	Recurrent respiratory papillomatosis
IFN-γ	Interferon-gamma
PD-L1	Programmed death-ligand 1
CTLA4	Cytotoxic lymphocyte antigen 4
IFS	Intervention-free survival
HPV	Human papillomavirus
TIGIT	T-cell immunoreceptor with Ig and ITIM domains
LAG3	Lymphocyte-activation gene 3
TIM3	T-cell immunoglobulin and mucin-domain containing-3
ICB	Immune checkpoint blockade
Tregs	Regulatory T-cells
HIV	Human immunodeficiency viruses
IL-4	Interleukin-4
TGF-β	Transforming growth factor-β
Th1	T helper type 1
