# Supplementary figures and images for: Local T-Cell Dysregulation and Immune Checkpoint Expression in Human Papillomavirus-Mediated Recurrent Respiratory Papillomatosis
Source: Cells. 2025 Jun 27;14(13):985. doi: 10.3390/cells14130985 (PMC12249130; doi:10.3390/cells14130985)

**a** Epithelium

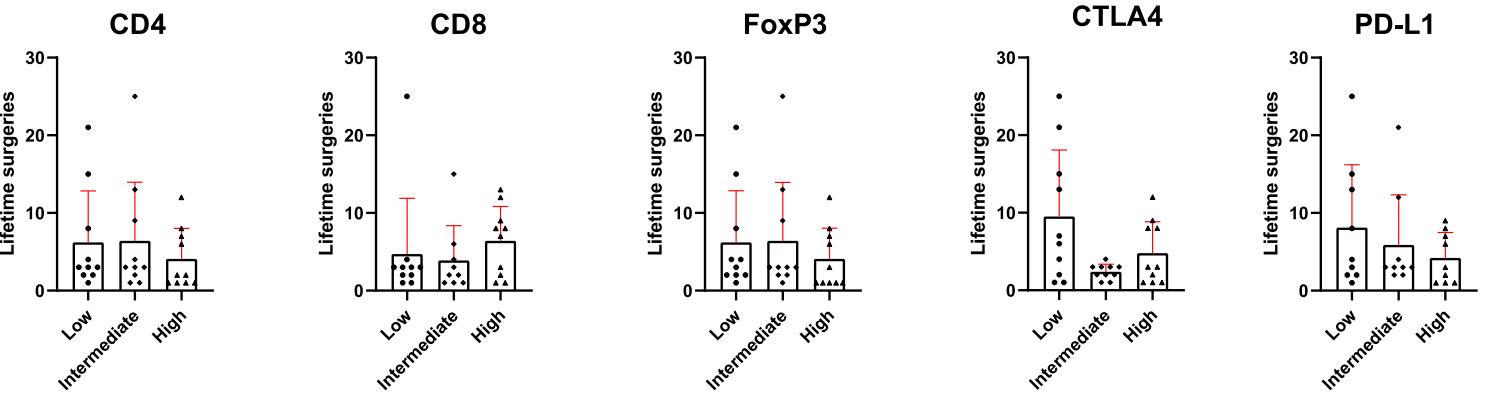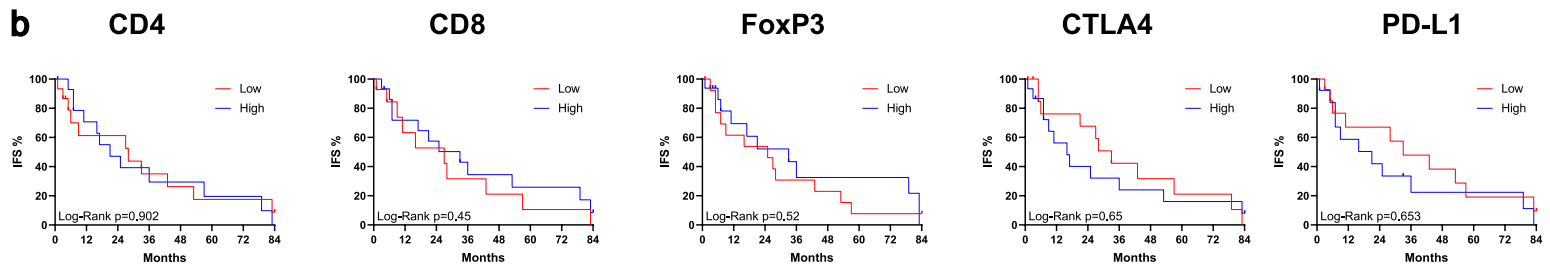

**c** Stroma

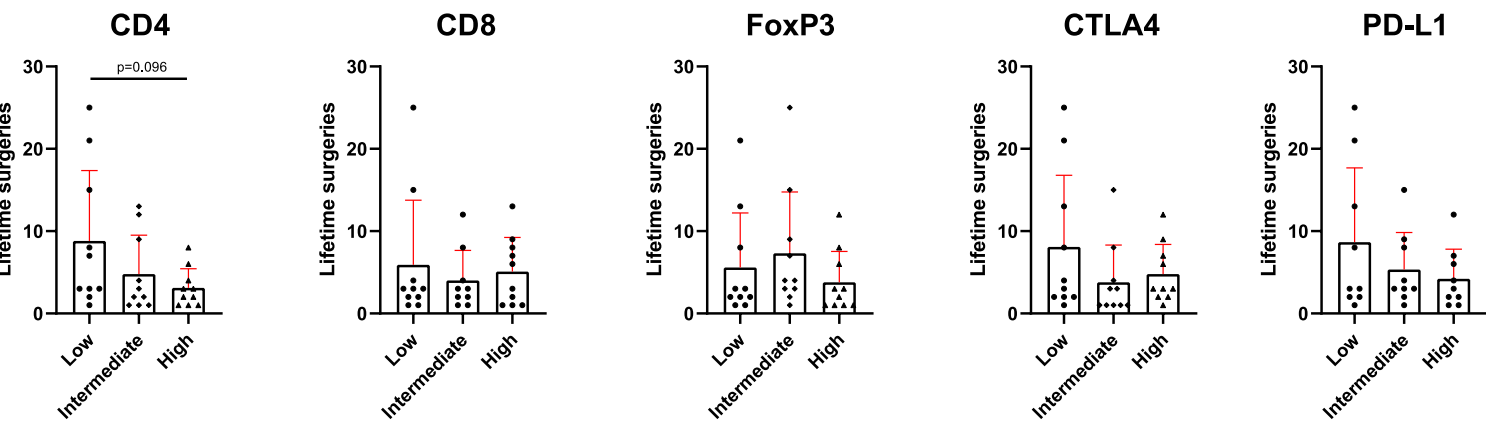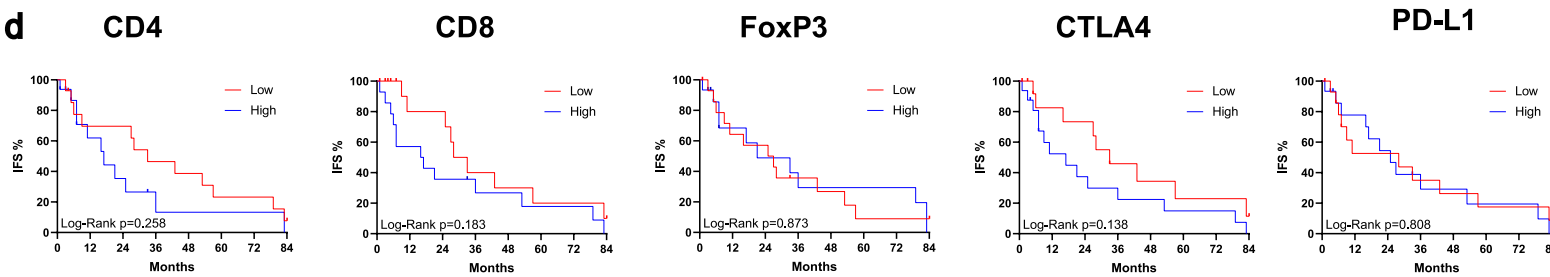

Supplement: Supplementary file 1 [file cells-14-00985-s001.zip › Sup Figure 1.pdf]
